# Cohort profile: the Ewha Birth and Growth Study

**DOI:** 10.4178/epih.e2021016

**Published:** 2021-02-22

**Authors:** Hye Ah Lee, Bohyun Park, Jungwon Min, Eun Jeong Choi, Ui Jeong Kim, Hyun Jin Park, Eun Ae Park, Su Jin Cho, Hae Soon Kim, Hwayoung Lee, Young Ju Kim, Young Sun Hong, Eui-Jung Kim, Eun Hee Ha, Hyesook Park

**Affiliations:** 1 Clinical Trial Center, Ewha Womans University Mokdong Hospital, Seoul, Korea; 2 National Cancer Control Institute, National Cancer Center, Goyang, Korea; 3 Department of Biomedical and Health Informatics, Children’s Hospital of Philadelphia, Philadelphia, PA, USA; 4 Department of Preventive Medicine, Ewha Womans University College of Medicine, Seoul, Korea; 5 Graduate Program in System Health Science and Engineering, Ewha Womans University, Seoul, Korea; 6 Department of Pediatrics, Ewha Womans University College of Medicine, Seoul, Korea; 7 Department of Anatomy, Ewha Womans University College of Medicine, Seoul, Korea; 8 Department of Obstetrics and Gynecology, Ewha Womans University College of Medicine, Seoul, Korea; 9 Department of Internal Medicine, Ewha Womans University College of Medicine, Seoul, Korea; 10 Department of Psychiatry, Ewha Womans University College of Medicine, Seoul, Korea; 11 Department of Occupational and Environmental Medicine, Ewha Womans University College of Medicine, Seoul, Korea

**Keywords:** Cohort studies, Child health, Cardiometabolic risk factors, Metabolic syndrome

## Abstract

With the introduction of life-course epidemiology, researchers realized the importance of identifying risk factors in early life to prevent chronic diseases. This led to the establishment of the Ewha Birth and Growth Study in 2001; the study is a prospective birth cohort designed to provide evidence of early life risk factors for a child’s growth and health. Participants were recruited from those who visited Ewha Womans University Mokdong Hospital (a tertiary hospital in southwest Seoul, Korea) for prenatal care at 24-28 weeks of gestation. In total, 891 mothers enrolled in this study between 2001 and 2006 and their offspring (n=940) were followed-up. Regular check-up examinations of offspring were conducted at 3 years, 5 years, and 7 years of age and every year thereafter. To consider age-related health issues, extensive data were collected using questionnaires and measurements. In 2021, the study subjects will reach 19 years of age, and we are planning a check-up examination for early adulthood. About 20 years have passed since the cohort data were collected, and we have published results on childhood health outcomes associated with prenatal and birth characteristics, genetic and epigenetic characteristics related to childhood metabolism, the effects of exposure to endocrine disruptors, and dietary patterns in childhood. Recently, we started reporting on topics related to adolescent health. The findings will facilitate identification of early life risk factors for chronic diseases and the development of interventions for diseases later in life.

## INTRODUCTION

According to a report from the World Health Organization, 41 million people die annually as a result of non-communicable diseases (NCDs), accounting for 71% of all deaths worldwide [1]. Similar results were reported in the 2012 Korean Burden of Disease Study [2]. In terms of disease burden, NCDs made a greater contribution to years lived with disability than to premature death [2]. As a result of the nature of NCDs, long-term treatment and care are required, which can affect the patients’ quality of life. In addition, the aging population will further increase the NCD burden. Therefore, amelioration and prevention of NCDs are important goals.

Longitudinal studies have suggested that the origin of NCDs in adulthood can be traced to early life [3,4]. The Bogalusa Heart Study began in 1972 in children aged 5-17 years and evaluated early-life risk factors for cardiovascular health. In that study, uric acid levels in childhood were predictive of high blood pressure in childhood and adulthood [5], and childhood blood pressures were reflected in adulthood [3]. The cumulative evidence for the long-term effects of early life on adult health implies the need for a birth cohort study [6]. In England, the Avon Longitudinal Study of Parents and Children began collecting genotype and environmental characteristics data on 13,761 pregnant women in the early 1990s, and collection of follow-up data is ongoing [7]. The Norwegian Mother and Child Cohort Study began recruiting pregnant women in 1999 and 2005 [8], and Project VIVA recruited pregnant women in 1999 to 2002 in eastern Massachusetts in the United States and in mid-2010 followed-up the offspring, who were then in their early teens [9]. Some epidemiological studies have reported results related to early-life risks for health later in life, but there were few relevant studies in Korea at that time. Accordingly, the Ewha Birth and Growth Study was designed to evaluate factors affecting growth and health in childhood, with a focus on cardiometabolic health. Here, we introduce the Ewha Birth and Growth Study and describe the main findings.

## STUDY PARTICIPANTS

### Description of the cohort and follow-up

Obstetricians, pediatricians, and experts in anatomy, epidemiology, and nutrition were involved in constructing the cohort. From September 2001 to June 2006, investigators recruited participants from those who visited the Ewha Womans University Mokdong Hospital (a tertiary hospital in southwest Seoul, Korea) for prenatal care at 24 weeks to 28 weeks of gestation. About 30% of all eligible women agreed to participate in the study and provide information at delivery [10]. A total of 891 women participated in this study, and their children (n= 940) were followed-up.

**Figure 1.**
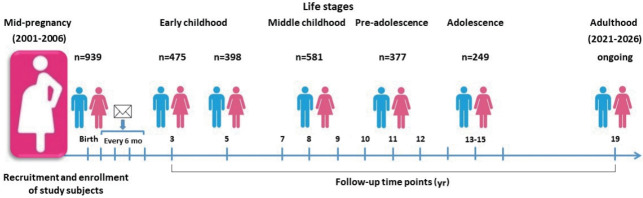
The Ewha Birth and Growth Study establishment and follow-up history.

Follow-up postal surveys were conducted at 6 months, 12 months, 18 months, and 24 months, and regular check-up examinations were conducted at 3 years, 5 years, and 7 years of age and annually thereafter (Figure 1). In 2015, check-up examinations at 13 years of age were begun. Check-up examinations at the ages of 14 years and 15 years were performed for subjects who did not receive check-ups at 13 years or 14 years of age. To overcome the reduction in sample size caused by the low follow-up rate, subjects born in the same hospital who visited the pediatric department at the age of 3 years were enrolled and followed-up. At the time of each follow-up, we informed the parents or guardians by telephone about the check-up examination. About half of the subjects were contacted, and about 75% participated in the regular check-up examinations. The children and their parents or guardians visited the hospital and the children fasted overnight for at least 8 hours. To minimize temporal and seasonal variations, we performed the follow-up examinations in the morning and at similar times of year. The check-up examinations involved anthropometric measurements, questionnaires, dietary surveys, and blood and urine sampling. Venous blood samples were collected into vacutainer tubes (plasma and serum). Blood and urine samples were stored at -70°C to -80°C until analysis. Individual dietary data were collected by trained dieticians during face-to-face interviews. In 2021, the subjects will reach 19 years of age, and we are planning a check-up examination for early adulthood. Table 1 lists the numbers of participants and anthropometric data across the follow-up visits.

### Ethics statement

The study protocol was approved by the Institutional Review Board (IRB) of Ewha Womans University Hospital. During follow-up, the research protocols were updated and reapproved by the IRB of Ewha Womans University Hospital. Because the research institute moved in 2019, a new IRB number was assigned (IRB No. SEUMC 2019-04-035). Written informed consent was obtained from all participants prior to the baseline survey. The parents or guardians of the subjects provided informed consent for participation in check-up examinations. Participants were informed that they could withdraw from the study at any time.

## MEASUREMENTS

We collected a range of data, including genotypes, from the cohort. Table 2 shows a list of measurements by follow-up time.

At birth, birth weight and body length were measured in the delivery room by trained nurses using digital scales. Gestational age was calculated based on the maternal report of the date of the last menstrual period and an ultrasound measurement performed by an obstetrician. Cord blood and placental tissue were sampled.

Anthropometric data were measured by trained researchers and nurses in the hospital. Height and weight were measured to the nearest 0.1 cm and 0.1 kg, respectively, using a stadiometer and calibrated scale, with the subject wearing light clothing and no shoes. Blood pressure was measured twice by trained researchers using an automatic device of the correct cuff size with participants resting in a stable position. Two measurements, taken within 5 minutes of each other, were averaged. Blood chemistry tests were performed for fasting blood glucose, lipid profile, and liver function enzymes, as well as a complete blood count.

Physical activity and sedentary behavior were assessed using questionnaires. Data on nutrient intake and food intake were collected by dietary survey, by 2 days of 24-hour recall for weekdays or using a food-frequency questionnaire (FFQ). The reproducibility and validity of the FFQ were acceptable [11]. As the subjects entered adolescence, the FFQ tool was modified by expert consensus, by adding 2 beverage items (e.g., coffee). The 24-hour dietary recall survey was conducted by the children’s guardians or parents (most frequently the mother). If children were attending kindergarten, the investigator attempted to contact their kindergarten teachers to collect accurate information. Nutrient intake was determined via CAN-pro version 3.0 (Korean Nutrition Society, Seoul, Korea). Dietary habits were assessed using a validated mini-dietary assessment tool [12]. To assess behavioral problems, we used the Korean version of the Child Behavior Checklist (K-CBCL) [13] and Conner’s Hyperactivity Rating Scale [14]. We also performed evaluations using the Children’s Depression Inventory [15], State-Trait Anxiety Inventory [16], and Trait Anxiety Inventory for Children [17].

At the follow-ups between 7 years and 9 years of age, for puberty evaluation, bone age and Tanner stage were evaluated by X-ray scans and the clinician’s assessment, respectively. Sex-hormone (luteinizing hormone, dehydroepiandrosterone, androstenedione, free testosterone, and free estradiol) concentrations were measured in blood samples. As the subjects entered adolescence, carotid intima-media thickness and endothelial dysfunction biomarkers were measured as indicators of atherosclerosis susceptibility. Carotid intima-media thickness was measured by a trained and certified sonographer.

For genotyping, genetic loci related to homocysteine metabolism (rs1801133 in methylenetetrahydrofolate reductase), the renin–angiotensin system (rs4341 in angiotensin-converting enzyme), and uric acid concentration (rs3825017 in solute carrier family 22 member 12 [*SLC22A12*] and rs16890979 in solute carrier family 2 facilitated glucose transporter member 9 [*SLC2A9*]) were identified. If stored cord blood was available, the methylation levels of proopiomelanocortin (POMC), the melanocortin 4 receptor (MC4R), and hepatocyte nuclear factor 4 alpha (HNF4a; associated with metabolic syndrome) were assayed in participants in check-up examinations at the ages of 7-9 years.

As part of a research project, the levels of endocrine disruptors—polychlorinated biphenyl, organochlorine pesticide, bisphenol A (BPA), and phthalates—in stored blood and urine samples were measured by Lab Frontier Co., Ltd. (Anyang, Korea).

## KEY FINDINGS

About 20 years have passed since the cohort data were collected, and we have published a broad spectrum of results. The findings from the Ewha Birth and Growth Study are listed in Supplemental Material 1 and some of the main findings are described below.

The Ewha Birth and Growth Study evaluated relationships between perinatal exposure, birth characteristics, and childhood health. Mendelian randomization revealed that the explainable homocysteine level during pregnancy due to genetic variants was causally related to birth weight [10]. Birth characteristics affect the variance in metabolic indicators in childhood. Regarding perinatal epigenetic effects, the methylation levels of POMC, MC4R, and HNF4a in cord blood were associated with triglyceride levels in children aged 7-9 years [18,19]. In addition, according to Park et al. [20,21], fetal characteristics are associated with elevated uric acid levels at 3 years of age, and high uric acid levels during the preschool period are associated with elevated blood pressure at the age of 7 years.

Hong et al. [22] reported that maternal micronutrient intake, as measured in blood, was persistently associated with growth during the first 3 years of life. Growth levels were linked to childhood blood pressure and pubertal development. The effect of angiotensin-converting enzyme genotype on blood pressure in childhood varies with postnatal growth [23]. Regarding pubertal development, longitudinal observations from birth to 7-9 years of age showed that weight Z-scores at 5 years of age differed significantly between subjects who did and did not experience early puberty. Postnatal weight changes from birth to 7-9 years of age were independently associated with early puberty. Notably, breastfeeding for 6 months or longer prevented early puberty [24].

Using dietary data collected at the ages of 3 years and 7 years, the relationship between macronutrient intake and lipid metabolism was evaluated [25]. During the 4-year follow-up, increased intake of carbohydrates had an unfavorable effect on triglyceride levels, and increased intake of fat, especially animal-based fat, increased low-density lipoprotein-cholesterol levels over time. Another study using FFQ data identified dietary patterns through principal component analysis and evaluated dietary behaviors associated with a healthier dietary pattern [11]. The dietary patterns—healthy intake, animal food intake, and snack intake—were similar at 7 years and 9 years of age. Changes in behavior that increased the consumption of milk or dairy products or encouraged the consumption of vegetables with every meal had favorable effects on changes in healthy dietary pattern scores over 2 years. These studies have shown that improved eating behavior can benefit childhood diet.

Taking advantage of the repeated measurement data, we evaluated candidate genotypes related to uric acid levels. The rs3825017 genotype of SLC22A12 was associated with a sex difference in uric acid levels during the first 10 years of life; however, the association was significant only in boys [26]. The consistency of the results at all time points supports the validity of the conclusions.

Other studies have assessed the health risks of exposure to endocrine disruptors. At routine low-dose exposure levels, exposure to endocrine disruptors affected metabolic-syndrome-related indicators [27,28], including insulin secretion [29], and was related to liver indices [30] and sex hormone levels [31]. Regarding the critical exposure period, the urinary BPA concentration tertile at 7-9 years, rather than 3-5 years, showed a dose-response relationship with liver enzyme levels at 10-13 years of age [30]. Using phthalate data measured repeatedly at ages 3-5 and 7-9, we evaluated the effect of long-term exposure to phthalates on metabolic indicators. Children with consistently high levels of exposure had high levels of triglycerides and homeostatic model assessment for insulin resistance scores, and low levels of high-density lipoprotein-cholesterol (HDL-c) [28]. Environmental exposure is associated with behavioral development as well as metabolic health in children. Using the K-CBCL data, we evaluated the effect of environmental tobacco smoke exposure (as assessed by cotinine levels) on behavioral problems [32]. High levels of cotinine at the age of 5 years were associated with higher total behavioral problem scores, a link that remained evident until the age of 9 years.

Recently, we started reporting on adolescent health-related research topics. A mediation analysis implied that, among the metabolic syndrome components, body mass index was directly related to elevated high-sensitivity C-reactive protein levels and indirectly via HDL-c to elevated intercellular adhesion molecule 1 levels [33]. The data collected were from young people, and further results are therefore expected to be published.

## STRENGTHS AND WEAKNESSES

The Ewha Birth and Growth cohort is a leading long-term follow-up study in Korea. The results from this cohort are consistent with previous findings in other countries. This cohort provides an opportunity to assess the effect of early life on later health, and the findings emphasize the need for interventions in early life to prevent subsequent NCDs.

Nonetheless, several limitations should be kept in mind. Although this cohort study consisted of a general population of children, the generalizability of the results is weakened by the hospital-based recruitment. Furthermore, the follow-up rate was low. Loss to follow-up resulting from moving house, changing telephone numbers, and withdrawal is inevitable in cohort studies. Loss to follow-up can lead to a decrease in the power for exploring the research hypothesis and the reliability of the results. A simulation study found that there was no significant bias until the follow-up loss was up to 60% based on the missing at random or missing completely at random mechanism [34]. As our cohort study considered various exposures in a typical setting and evaluated the outcome prospectively, it is thought that the loss in follow-up was random. Thus, the resulting loss of follow-up measures may affect the reliability of the results, but the direction of the association was consistent with other studies. Finally, the cohort size is smaller than that of other birth cohorts, but we increased the power of the statistical tests by performing repeated measurements.

In our experience, the nature of a prospective cohort study entails long-term follow-up and it is necessary to build a trustful relationship between participants and researchers to prevent follow-up loss and to maintain participation. Long-term follow-up involves not only financial funding, but also maintenance and management of personnel, so a large amount of effort is required to maintain the cohort. In addition, to expand and utilize cohort resources, there is a need to be concerned with health issues along with social trends and to explore potential research questions.

## DATA ACCESSIBILITY

The cohort data are not freely available, but the Ewha Birth and Growth Study team welcomes collaborations with other researchers. For further information, contact Dr. Park (the corresponding author).

## Figures and Tables

**Table 1.** Summary of the structure of the Ewha Birth and Growth Study^1^

Characteristics	At birth	Follow-up examinations (yr)								
		3	5	7	8	9	10	11	12	13-15
Boys										
No. of subjects	487	236	202	161	199	200	123	133	116	122
Height (cm)	49.4±2.4	97.8±4.5	112.2±5.3	124.4±5.7	130.3±5.6	135.0±5.7	139.1±6.0	145.8±6.1	152.5±7.8	162.8±7.0
Z-score of height	-0.2±1.3	-0.5±1.2	-0.2±1.2	-0.2±1.1	-0.1±1.1	-0.2±1.0	-0.5±1.0	-0.3±0.9	-0.3±1.1	0.0±0.9
Weight (kg)	3.2±0.5	14.9±2.0	20.1±3.4	25.3±4.9	29.1±6.3	32.8±6.9	35.5±7.6	41.2±9.4	47.8±11.0	56.0±12.3
Z-score of weight	-0.3±1.2	-0.7±1.3	-0.2±1.3	-0.4±1.2	-0.3±1.2	-0.3±1.1	-0.4±1.1	-0.3±1.1	-0.1±1.1	0.1±1.1
BMI (kg/m^2^)	13.2±1.3	15.5±1.4	15.9±1.8	16.2±2.2	17.0±2.7	17.8±2.8	18.2±3.1	19.2±3.2	20.4±3.4	21.1±3.8
Overweight^2^	-	12 (5.2)	13 (6.4)	11 (6.9)	14 (7.1)	16 (8.1)	12 (9.8)	11 (8.3)	8 (7.7)	5 (5.1)
Obesity^3^	-	13 (5.7)	16 (7.9)	11 (6.9)	18 (9.1)	16 (8.1)	7 (5.7)	10 (7.5)	13 (12.5)	12 (12.1)
SBP (mmHg)	65.8±8.1	93.8±10.5	93.8±10.5	100.8±9.8	106.0±10.8	107.7±10.9	110.2±11.7	111.8±10.0	110.0±13.5	111.8±11.8
DBP (mmHg)	39.2±8.0	60.2±9.6	59.2±6.6	58.4±6.7	61.1±6.9	63.1±6.6	69.8±8.9	69.6±8.4	68.7±8.3	68.3±9.2
High BP^4^	-	-	-	6 (3.8)	4 (2.0)	7 (3.5)	4 (3.3)	5 (3.8)	4 (3.5)	3 (2.5)
Girls										
No. of subjects	452	239	196	201	183	199	113	143	113	127
Height (cm)	48.7±2.7	96.1±4.2	111.5±4.7	122.8±4.9	129.9±5.0	134.7±5.7	139.2±5.7	147.0±6.3	153.6±6.1	158.2±5.4
Z-score of height	-0.3±1.4	-0.7±1.1	0.0±1.1	-0.2±1.0	0.0±0.9	-0.2±1.0	-0.5±0.9	-0.3±1.0	0.0±1.0	0.1±1.0
Weight (kg)	3.1±0.5	14.3±1.7	19.4±2.8	23.9±4.1	28.2±5.3	31.4±5.9	34.8±7.2	40.3±7.6	46.3±8.5	51.1±8.3
Z-score of weight	-0.3±1.3	-0.7±1.2	-0.2±1.2	-0.4±1.1	-0.2±1.0	-0.3±1.0	-0.4±1.0	-0.3±1.0	0.0±1.0	0.1±1.0
BMI (kg/m^2^)	13.1±1.5	15.4±1.3	15.5±1.5	15.8±2.1	16.6±2.5	17.2±2.4	17.8±2.7	18.6±2.8	19.6±3.0	20.4±2.9
Overweight^2^	-	19 (8.2)	11 (5.6)	8 (4.0)	13 (7.1)	19 (9.6)	9 (8.0)	7 (4.9)	13 (13.8)	15 (11.8)
Obesity^3^	-	11 (4.7)	8 (4.1)	10 (5.0)	9 (4.9)	7 (3.5)	8 (7.1)	9 (6.3)	7 (7.5)	9 (7.1)
SBP (mmHg)	68.2±9.4	93.5±9.5	98.5±9.4	99.1±10.1	103.1±11.4	105.6±10.6	109.8±10.8	111.6±11.0	108.9±11.5	108.7±11.1
DBP (mmHg)	39.3±8.0	60.3±8.9	59.3±6.4	59.2±6.8	60.5±7.3	62.4±7.4	69.5±8.0	71.0±9.3	68.5±8.8	66.9±7.9
High BP^4^	-	-	-	1 (0.5)	2 (1.1)	5 (2.5)	1 (0.9)	2 (1.4)	0 (0.0)	2 (1.6)

Values are presented as mean±standard deviation or number (%). The sex- and age-specific Z-scores of height and weight were calculated based on the 2017 Korean Children and Adolescent National Growth Charts; Overweight and obesity were defined based on the 2017 Korean Children and Adolescent National Growth Charts; High BP was defined based on the 2007 Korean Children and Adolescent National Growth Charts. BMI, body mass index; SBP, systolic blood pressure; DBP, diastolic blood pressure; BP, blood pressure.

1 Numbers vary as a result of missing data.

2 Overweight: 85th ≤ BMI < 95th percentile.

3 Obesity: BMI ≥ 95th percentile or BMI ≥25.0 kg/m^2^.

4 High BP: SBP or DBP ≥ 90th percentile or SBP/DBP ≥120/80 mmHg.

**Table 2.** Summary of measured phenotypes in the Ewha Birth and Growth Study

Phenotype			At birth	Follow-up examinations												
				6 mo^1^	12 mo^1^	18 mo^1^	24 mo^1^	3 yr	5 yr	7 yr	8 yr	9 yr	10 yr	11 yr	12 yr	13-15 yr
Questionnaires																
	Demographics			O^2^	O^2^	O^2^	O^2^	O	O	O	O	O	O	O	O	O
	Breastfeeding			O^2^	O^2^	O^2^	O^2^	O	O	O	O	O	-	-	-	-
	Physical activity			O^2^	O^2^	O^2^	O^2^	O	O	O	O	O	-	O	O	O
	Parental disease history (HTN, DM, stroke, etc.)			-	-	-	-	O	O	O	O	O	-	O	O	O
	Secondhand smoking			O^2^	O^2^	O^2^	O^2^	O	O	O	O	O	O	O	O	O
	K-CBCL			-	-	-	-	-	O	O	-	O	-	O^2^	O	-
	Conners Hyperactivity Rating Scale			-	-	-	-	-	O	O	-	O	-	O	O	-
	ADHD			-	-	-	-	-	O	O	-	O	-	O	O	-
	CDI, STAI, and TAIC			-	-	-	-	-	-	-	-	O^2^	-	O^2^	O^2^	-
	Pubertal status (Tanner stage)			-	-	-	-	-	-	O	O	O	O	O	O	O
Nutrition survey																
	Diet (24-hr recall)			-	-	-	-	O	O^2^	O	-	-	-	-	-	-
	Diet (FFQ)			-	-	-	-	-	O^2^	O	O	O	O	O	O	O
	Eating behaviors			-	-	-	-	O	-	O	O	O	O	O	O	O
Physical examination																
	Anthropometric			O^2^	O^2^	O^2^	O^2^	O	O	O	O	O	O	O	O	O
	Blood pressure			-	-	-	-	O	O	O	O	O	O	O	O	O
	Hand-grip strength			-	-	-	-	-	-	O	O	O	-	-	-	-
	Bone mineral density			-	-	-	-	-	O^2^	-	O^2^	O^2^	-	-	-	-
	Body composition (muscle, % body fat, etc.)			-	-	-	-	O^2^	O	O^2^	O	O	-	-	-	-
	Bone age			-	-	-	-	-	-	O^2^	O^2^	O^2^	-	-	-	-
	Carotid intima-media thickness			-	-	-	-	-	-	-	-	-	-	-	-	O
Blood																
	Chemistries			-	-	-	-	O	O	O	O	O	O	O	O	O
	Liver enzymes (gamma-glutamyl transferase)			-	-	-	-	-	-	-	-	-	O	O	O	O
	Persistent organic pollutants			-	-	-	-	-	-	O^2^	O^2^	O^2^	-	-	-	-
	Lipids (total cholesterol, triglyceride, high-density lipoprotein cholesterol)			-	-	-	-	O	O	O	O	O	O	O	O	O
	Glucose			-	-	-	-	O	O	O	O	O	O	O	O	O
	Insulin			-	-	-	-	O^2^	O	O	O	O	O^2^	O^2^	-	O
	Uric acid			-	-	-	-	O^2^	O	O	O	O	O	O	O	O
	Endothelial dysfunction (ICAM-1, VCAM-1)			-	-	-	-	-	-	-	-	-	-	-	-	O
	Inflammatory biomarkers (hs-CRP, IL-6)			-	-	-	-	O^2^	O	O	O	O	-	-	-	O
	Sex hormones (luteinizing hormone, DHEA, androstenedione, testosterone, estradiol)			-	-	-	-	-	-	O	O^2^	O^2^	-	-	-	-
	Thyroid-stimulating hormone			-	-	-	-	-	-	O^2^	O^2^	O^2^	O	O	O	O
	Free thyroxine (fT_4_)			-	-	-	-	-	-	O^2^	O^2^	O^2^	O^2^	O^2^	O^2^	-
	25-Hydroxyvitamin D			-	-	-	-	-	-	O	O	O	-	-	-	-
	Oxidative stress															
		8-OHdG		-	-	-	-	O^2^	O^2^	O^2^	-	-	-	-	-	-
		MDA		-	-	-	-	O^2^	O^2^	O^2^	-	-	-	-	-	-
	Vitamins															
		Vitamin C		-	-	-	-	O^2^	-	-	-	-	-	-	-	-
		Retinol		-	-	-	-	O^2^	-	-	-	-	-	-	-	-
		Tocopherol		-	-	-	-	O^2^	-	-	-	-	-	-	-	-
		Carotene		-	-	-	-	O^2^	-	-	-	-	-	-	-	-
	Methylation															
		Proopiomelanocortin		-	-	-	-	-	-	O^2^	O^2^	O^2^	-	-	-	-
		Hepatocyte nuclear factor 4 alpha		-	-	-	-	-	-	O^2^	O^2^	O^2^	-	-	-	-
		Melanocortin 4 receptor		-	-	-	-	-	-	O^2^	O^2^	O^2^	-	-	-	-
Urine																
	Cotinine			-	-	-	-	O^2^	O	O	-	-	-	-	-	-
	Bisphenol A			-	-	-	-	O	O	O	O^2^	O	-	-	-	-
	Phthalate metabolites			-	-	-	-	O^2^	O^2^	O^2^	O^2^	O^2^	-	-	-	-
Single-nucleotide polymorphisms			Glutathione S-transferase T1													
			Glutathione S-transferase M1													
			Angiotensin-converting enzyme rs4341													
			Paraoxonase 1 rs662													
			Myeloperoxidase rs2333227													
			Peroxisome proliferator-activated receptor gamma rs1801282													
			Fat mass and obesity‐associated rs9939609													
			Solute carrier family 2 facilitated glucose transporter member 9 rs16890979													
			Solute carrier family 22 member 12 rs3825017													

HTN, hypertension; DM, diabetes mellitus; K-CBCL, Korean-Childhood Behavioral Checklist; ADHD, attention deficit hyperactivity disorder; CDI, Children’s Depression Inventory; STAI, State-Trait Anxiety Inventory; TAIC, Trait Anxiety Inventory for Children; FFQ, food frequency questionnaire; ICAM-1, intercellular adhesion molecule; VCAM-1, vascular cell adhesion molecule 1; hs-CRP, high-sensitivity C-reactive protein; IL, Interleukin; DHEA, dehydroepiandrosterone; MDA, malondialdehyde, 8-OHdG, 8-hydroxy-2′-deoxyguanosine.

1 Follow-up examinations were conducted by mail.

2 Only a portion of the subjects was measured.

## SUPPLEMENTARY MATERIALS

Supplementary Material 1. Research hypothesis

## References

[1] World Health Organization (2018). Noncommunicable diseases. https://www.who.int/news-room/fact-sheets/detail/noncommunicable-diseases.

[2] Yoon J, Seo H, Oh IH, Yoon SJ (2016). The non-communicable disease burden in Korea: findings from the 2012 Korean Burden of Disease study. J Korean Med Sci.

[3] Du T, Fernandez C, Barshop R, Chen W, Urbina EM, Bazzano LA (2019). 2017 Pediatric hypertension guidelines improve prediction of adult cardiovascular outcomes. Hypertension.

[4] Katzmarzyk PT, Pérusse L, Malina RM, Bergeron J, Després JP, Bouchard C (2001). Stability of indicators of the metabolic syndrome from childhood and adolescence to young adulthood: the Québec Family Study. J Clin Epidemiol.

[5] Alper AB, Chen W, Yau L, Srinivasan SR, Berenson GS, Hamm LL (2005). Childhood uric acid predicts adult blood pressure: the Bogalusa Heart Study. Hypertension.

[6] Terry MB, Flom J, Tehranifar P, Susser E (2009). The role of birth cohorts in studies of adult health: the New York women’s birth cohort. Paediatr Perinat Epidemiol.

[7] Fraser A, Macdonald-Wallis C, Tilling K, Boyd A, Golding J, Davey Smith G (2013). Cohort profile: the Avon Longitudinal Study of Parents and Children: ALSPAC mothers cohort. Int J Epidemiol.

[8] Magnus P, Birke C, Vejrup K, Haugan A, Alsaker E, Daltveit AK (2016). Cohort profile update: the Norwegian Mother and Child Cohort Study (MoBa). Int J Epidemiol.

[9] Oken E, Baccarelli AA, Gold DR, Kleinman KP, Litonjua AA, De Meo D (2015). Cohort profile: project viva. Int J Epidemiol.

[10] Lee HA, Park EA, Cho SJ, Kim HS, Kim YJ, Lee H (2013). Mendelian randomization analysis of the effect of maternal homocysteine during pregnancy, as represented by maternal MTHFR C677T genotype, on birth weight. J Epidemiol.

[11] Lee HA, Hwang HJ, Oh SY, Park EA, Cho SJ, Kim HS (2016). Which diet-related behaviors in childhood influence a healthier dietary pattern? From the Ewha Birth and Growth Cohort. Nutrients.

[12] Kim WY, Cho MS, Lee HS (2003). Development and validation of mini dietary assessment index for Koreans. Korean J Nutr.

[13] Oh KJ, Lee HR, Hong KE, Ha EH (1997). Manual for K-CBCL: Korean Child Behavior Checklist.

[14] Park EH, So YK, Choi NK, Kim SJ, Noh JS, Ko YJ (2003). The reliability and validity of Korean Conners Parent and Teacher Rating scale. Korean J Child Adol Psychiatr.

[15] Cho SC, Lee YS (1990). Development of the Korean form of the Kovacs’ Children’s Depression Inventory. J Korean Neuropsychiatr Assoc.

[16] Kim JT, Shin DK (1978). A study based on the standardization of the STAI for Korea. New Med J.

[17] Cho SC, Choi JS (1989). Development of the Korean version of State-Trait Anxiety Inventory for Children. Seoul J Psychiatry.

[18] Yoo JY, Lee S, Lee HA, Park H, Park YJ, Ha EH (2014). Can proopiomelanocortin methylation be used as an early predictor of metabolic syndrome?. Diabetes Care.

[19] Kwon EJ, Lee HA, You YA, Yoo JY, Park H, Park EA (2019). MC4R and HNF4α promoter methylation at birth contribute to triglyceride levels in childhood: a prospective cohort study. Medicine (Baltimore).

[20] Park B, Park E, Cho SJ, Kim Y, Lee H, Min J (2009). The association between fetal and postnatal growth status and serum levels of uric acid in children at 3 years of age. Am J Hypertens.

[21] Park B, Lee HA, Lee SH, Park BM, Park EA, Kim HS (2017). Association between serum levels of uric acid and blood pressure tracking in childhood. Am J Hypertens.

[22] Hong J, Lee HA, Park EA, Kim YJ, Lee H, Park BH (2014). Association of mid-pregnancy antioxidative vitamin and oxidative stress levels with infant growth during the first 3 years of life. Food Nutr Res.

[23] Min J, Kim YJ, Lee H, Park EA, Cho SJ, Hong YM (2012). Is the association between ACE genes and blood pressure mediated by postnatal growth during the first 3 years?. Early Hum Dev.

[24] Lee HA, Kim YJ, Lee H, Gwak HS, Hong YS, Kim HS (2015). The preventive effect of breast-feeding for longer than 6 months on early pubertal development among children aged 7-9 years in Korea. Public Health Nutr.

[25] Lee HA, Hwang HJ, Oh SY, Park EA, Cho SJ, Kim HS (2018). The differential effects of changes in individual macronutrient intake on changes in lipid concentrations during childhood: from the Ewha Birth & Growth Cohort. Clin Nutr.

[26] Lee HA, Park BH, Park EA, Cho SJ, Kim HS, Park H (2018). Long-term effects of the SLC2A9 G844A and SLC22A12 C246T variants on serum uric acid concentrations in children. BMC Pediatr.

[27] Lee HA, Park SH, Hong YS, Ha EH, Park H (2016). The effect of exposure to persistent organic pollutants on metabolic health among Korean children during a 1-year follow-up. Int J Environ Res Public Health.

[28] Han H, Lee HA, Park B, Park B, Hong YS, Ha EH (2019). Associations of phthalate exposure with lipid levels and insulin sensitivity index in children: a prospective cohort study. Sci Total Environ.

[29] Park SH, Ha E, Hong YS, Park H (2016). Serum levels of persistent organic pollutants and insulin secretion among children age 7-9 years: a prospective cohort study. Environ Health Perspect.

[30] Lee S, Lee HA, Park B, Han H, Park BH, Oh SY (2018). A prospective cohort study of the association between bisphenol A exposure and the serum levels of liver enzymes in children. Environ Res.

[31] Lee HA, Kim YJ, Lee H, Gwak HS, Park EA, Cho SJ (2013). Effect of urinary bisphenol A on androgenic hormones and insulin resistance in preadolescent girls: a pilot study from the Ewha Birth & Growth Cohort. Int J Environ Res Public Health.

[32] Park B, Park B, Kim EJ, Kim YJ, Lee H, Ha EH (2020). Longitudinal association between environmental tobacco smoke exposure and behavioral problems in children from ages 5 to 9. Sci Total Environ.

[33] Lee HA, Choi EJ, Park B, Lee H, Hong YS, Kim HS (2020). The association between metabolic components and markers of inflammatory and endothelial dysfunction in adolescents, based on the Ewha Birth and Growth Cohort Study. PLoS One.

[34] Kristman V, Manno M, Côté P (2004). Loss to follow-up in cohort studies: how much is too much?. Eur J Epidemiol.

