# Supplementary material for: Cohort profile: the Ewha Birth and Growth Study
Source: Epidemiol Health. 2021 Feb 22;43:e2021016. doi: 10.4178/epih.e2021016 (PMC8060523; doi:10.4178/epih.e2021016)
Supplement: Supplementary Material 1. — Research hypothesis [file epih-43-e2021016-suppl1.docx]

Supplementary Material 1. Research hypothesis

| Category | Independent factors | Outcomes | Ref | Summary |
| --- | --- | --- | --- | --- |
| Birthweight & growth | Maternal serum levels of vitamins C and E during the second trimester | Birthweight and length | Eur J Clin Nutr. 2004; 58(10): 1365-71. | Maternal serum vitamin C levels during the second trimester were positively correlated with birthweight and length in full-term babies. |
|  | Oxidative stress in midterm pregnancy | Postnatal growth | Epidemiol Health. 2004; 26(1): 17-26. | The fourth quartile of malondialdehyde levels during mid-pregnancy was associated with low body weight at birth, 6 and 12 months. |
|  | Myeloperoxidase polymorphism, serum vitamin C level | Maternal oxidative stress and birthweight | J. Toxicol. Pub. Health 2004; 20(3): 187-93 | Urine 8-hydroxy-2'-deoxyguanosine and birthweight were associated with myeloperoxidase (MPO) genetic polymorphism, depending on maternal vitamin C levels. |
|  | Maternal serum folate and the MTHFR Polymorphisms | Infant birthweight | Molecular & cellular toxicology.  2005; 1(2): 130-6 | The birthweights of children with mothers with the MTHFR *CC* genotype and folate deficiency were relatively low. |
|  | Paraoxonase gene polymorphism and vitamin levels during pregnancy | Maternal oxidative stress and neonatal birthweights | Reprod Toxicol. 2006; 22(3): 418-24. | Oxidative stress during pregnancy was affected by serum vitamins C and E, and PON polymorphism. |
|  | Maternal micronutrient levels and oxidative stress | Growth during the first year of life | Public Health Nutr. 2008; 11(10): 998-1005. | Maternal serum vitamin C values during pregnancy were positively associated with the growth of offspring during the first year of life. The effect of folate status during pregnancy on the growth of offspring was not significant after adjusting for the covariates. |
|  | Oxidative stress (from mid pregnancy to delivery) | Birth size | Placenta. 2009; 30(5): 418-23. | There was no correlation between levels of oxidative stress in urine during pregnancy and in the placenta during delivery, but their interaction effects on birth size were significant. |
|  | Maternal MTHFR C677T genotype | Birthweight | J Epidemiol. 2013; 23(5): 371-5. | Maternal homocysteine levels explained by the MTHFR C677T genotype were negatively related to birthweight. |
|  | Maternal micronutrient levels and oxidative stress | Growth during the first 3 years of life | Food Nutr Res. 2014;58. | Maternal serum vitamin A and C values during pregnancy were positively associated with the growth of offspring during the first 3 years of life. |
|  | Breast-feeding | Pubertal development | Public Health Nutr. 2015; 18(18): 3300-7. | The patterns of weight change from birth to follow-up varied with early puberty development. Beneficial effects of breast-feeding on early puberty development have been found. |
|  | Adrenal androgen levels | Bone age advancement | J Korean Med Sci. 2017; 32(6): 968-73. | Bone age (BA) advancement was positively associated with androstenedione and testosterone levels regardless of body mass index. |

Supplemental Table 1. Research hypothesis (continued).

| Category | Independent factors | Outcomes | Ref | Summary |
| --- | --- | --- | --- | --- |
| Birthweight & growth | MKRN3 and LIN28B gene polymorphisms | Precocious puberty in Korean boys and girls | BMC Genet. 2018; 19(1): 47. | Boys with TT alleles in rs12441827 had about four-times greater risk for precocious puberty compared to C allele carriers. |
| Cardiometabolic risk | Intrauterine growth variables | Neonatal blood pressure | Korean Journal of Pediatrics. 2006; 49(9): 966-71. | Birthweight, height, and head circumference were positively correlated with neonatal blood pressure even after adjusting for gestational age, baby sex, and maternal blood pressure. |
|  | Birthweight and postnatal catch-up growth | Blood pressure | J Hum Hypertens. 2007; 21(11): 868-74. | For blood pressure in early life, birthweight is not directly related to blood pressure, but conditional weight gain and current weight seem to have an effect on blood pressure. |
|  | Maternal body weight change | Obesity | J Korean Soc Matern Child Health. 2011; 15(1): 82-91. | Low pre-pregnancy body mass index and low gestational weight gain are associated with high weight of offspring at 3 years old. |
|  | Angiotensin-converting enzyme (ACE) gene | Blood pressure | Early Hum Dev. 2012; 88(6): 425-9. | The effect of angiotensin-converting enzyme (ACE) genotype on blood pressure was mediated by postnatal growth during the first 3 years. |
|  | 25-Hydroxyvitamin D | Adiposity indices | J Pediatr Endocrinol Metab. 2013; 26(9-10): 849-54. | The level of 25(OH)D was inversely associated with body mass index, waist circumference, and percent body fat even after adjusting for covariates. |
|  | Urinary bisphenol A | Androgenic hormones and insulin resistance | Int J Environ Res Public Health. 2013; 10(11): 5737-49. | Exposure to relatively high levels of BPA increased androgen hormone levels in pre-pubescent girls, and the adverse effects of BPA on endocrine metabolism were consistent over 1 year. |
|  | Proopiomelanocortin methylation | Metabolic syndrome | Diabetes Care. 2014; 37(3): 734-9. | People with high propiomelanocortin (POMC) methylation in cord blood had higher triglyceride and insulin levels in childhood compared to others. POMC methylation was also related to birthweight. |
|  | 25-Hydroxyvitamin D | Metabolic syndrome | Nutrition. 2015; 31(11-12): 1324-7. | Serum 25(OH)D levels were negatively associated with serum triacylglycerol levels in prepubertal children. Other metabolic components are not related to 25(OH)D levels. |
|  | Persistent organic pollutants | Metabolic health changes | Int J Environ Res Public Health. 2016; 13(3): 270. | The concentrations of marker polychlorinated biphenyls (PCBs) were significantly associated with increased change in diastolic blood pressure (BP) and triglyceride levels during a 1-year follow-up. |
|  | Thyroid hormones | Blood pressure | J Pediatr Endocrinol Metab. 2016; 29(4): 459-64. | Serum thyroid hormones (thyroid-stimulating hormone and free thyroxine) are not independently associated with increased blood pressure in euthyroid preadolescents. |

Supplemental Table 1. Research hypothesis (continued).

| Category | Independent factors | Outcomes | Ref | Summary |
| --- | --- | --- | --- | --- |
| Cardiometabolic risk | Persistent organic pollutants | Insulin secretion | Environ Health Perspect. 2016; 124(12): 1924-30. | Subjects in the highest tertile showed a decreased homeostatic model assessment of beta-cell function (HOMA-β) compared to subjects in the lowest tertile. The trend was maintained over the two-year follow-up period. |
|  | Levels of uric acid | Blood pressure tracking | Am J Hypertens. 2017; 30(7): 713-8. | Uric acid levels at 3 years of age continued to affect subsequent BP. Subjects with high uric acid levels at both 3 and 5 years of age had the highest SBP at 7 years of age. |
|  | DNA methylations of POMC, MC4R, and HNF4A | Metabolic profiles in blood (7–9 years) | BMC Pediatr. 2018; 18(1): 121. | The cross-sectional association between methylation and lipid profile was evaluated. A significant association was found between propiomelanocortin (POMC) methylation and HDL-c levels, and HNF4A methylation and TC levels. |
|  | Macronutrient intake | Lipid concentrations | Clin Nutr. 2018; 37(3): 1027-33. | During the 4-year follow-up, a 1% increase in carbohydrate intake increased TG by 0.59 mg/dL compared to baseline (at 3 years old). |
|  | Urinary phthalate | Lipid and insulin levels | Sci Total Environ. 2019; 662: 714-21. | Levels of phthalate metabolites were higher at preschool age than at school age. Some phthalate metabolites were associated with increased TG and HOMA-IR levels at 3–5 years of age and 7–9 years of age. |
|  | MC4R and HNF4α methylation | Lipid concentrations | Medicine (Baltimore). 2019; 98(28): e16424. | The methylation statuses of MC4R and HNF4a at birth were associated with triglyceride levels in childhood. |
|  | Pre-and postnatal growth | Longitudinal trends in serum uric acid levels and BP | BMC Pediatr. 2020; 20(1): 23. | Based on follow-up from the age of 3–7 years, children who experienced low birthweight (LBW) and catch-up growth (CUG) had higher uric acid levels and greater changes in SBP over 4 years compared to normal-birthweight children. |
| Cardiovascular-disease-related markers | Pre-and postnatal growth | Uric acid level | Am J Hypertens. 2009; 22(4): 403-8. | Preterm birth and a subsequent high postnatal weight gain (during the first 3 years) were associated with increased serum uric acid levels at 3 years of age. |
|  | Folate and adiposity | Homocysteine | Nutr Res Pract. 2016; 10(1): 74-80. | Homocysteine levels at the age of 3 had a positive association with adiposity index and a negative association with folate levels. The combined effect of high adiposity and low folate levels was associated with high homocysteine levels. |

Supplemental Table 1. Research hypothesis (continued).

| Category | Independent factors | Outcomes | Ref | Summary |
| --- | --- | --- | --- | --- |
| Cardiovascular-disease-related markers | SLC2A9 G844A and SLC22A12 C246T polymorphisms | Longitudinal trends in serum uric acid levels | BMC Pediatr. 2018; 18(1): 296. | Boys with the T allele of rs3825017 (SLC22A12 c. 246C > T) had higher concentrations than their counterparts regardless of the time of follow-up. The rs16890979 genotypes (SLC2A9 c. 844G > A) were not significantly associated with serum UA concentration in either sex. |
|  | Prenatal growth status | Serum uric acid, cystatin C, blood urea nitrogen, creatinine levels, and estimated glomerular filtration rate | J Korean Med Sci. 2019; 34(25): e174. | As birthweight decreased, uric acid and blood urea nitrogen (BUN) at ages 7–12 tended to increase. In addition, Cystatin C tended to increase as gestational weeks decreased. |
|  | Continuous metabolic syndrome scores (cMets) | hsCRP, ICAM-1, and VCAM-1 | PLoS One. 2020; 15(5): e0233469. | An increase of one SD in the cMets score was related to an unfavorable inflammatory status and endothelial dysfunction in adolescents (aged 13–15 years). |
|  | Dietary zinc and obesity | Uric acid levels | Nutr Res Pract. 2020; 14(4): 365-73. | The dietary zinc intake level at 3 years of age and the BMI level at 7 years of age were related to uric acid levels in children at 7 years of age. |
| Others | Folate and homocysteine levels during pregnancy | DNA methylation in human placenta | J Peve Med Public Health. 2005; 38(4): 437-42. | Maternal folate and homocysteine levels along with the methylenetetrahydrofolate reductase (MTHFR) 677 genetic polymorphism during pregnancy affect DNA methylation in the human placenta. |
|  | Intrauterine oxidative stress and antioxidant vitamins | Childhood behavioral development at 4 years of age | J Korean Neuropsychiatr Assoc. 2009; 48(3): 153-9. | Maternal vitamins and oxidative stress during pregnancy were associated with the development of behavior in offspring. |
|  | Birthweight and body weight at 3 yrs | Respiratory illness | J Prev Med Public Health. 2010; 43(5): 369-76. | Children in the lowest birthweight tertile (aOR = 3.97, 95% CI = 0.94-16.68) or highest BMI tertile (aOR = 3.68, 95% CI = 1.24-10.95) at 3 years of age were at increased risk of chronic respiratory illness. |
|  | Environmental tobacco smoke exposure and glutathione s-transferase polymorphism | Childhood behavioral development | J Korean Neuropsychiatr Assoc. 2011; 50: 465-75. | Environmental tobacco smoke (ETS) exposure during mid-pregnancy based on urine cotinine levels, not ETS during early childhood, had a significant association with increased total scores and externalizing problem scores of K-CBCL. This did not depend on glutathione S-transferase polymorphisms. |

Supplemental Table 1. Research hypothesis (continued).

| Category | Independent factors | Outcomes | Ref | Summary |
| --- | --- | --- | --- | --- |
| Others | Early life risk factors | Bone mineral density (BMD) at 5 years old | J Korean Soc Matern Child Health. 2014; 18(2): 186-95. | Advanced maternal age (≥ 35 years) was independently associated with offspring low bone mineral density (< 25th percentile), even after adjusting for sex, sedentary activities, and household income (OR 3.7, 95% CI 1.6~8.9). |
|  | Serum levels of polychlorinated biphenyls (PCBs) and organochlorine pesticides (OCPs) | Persistent organic pollutants in blood (POPs) | Environ Sci Pollut Res Int. 2016; 23(4): 3536-47. | We analyzed a total of 51 persistent organic pollutants (POPs) in children aged 7–9 years. Serum concentrations of total polychlorinated biphenyls (PCBs) increased with age, but did not vary by sex. |
|  | Obesity index | Thyroid hormones | Korean journal of obesity. 2015; 24(4): 212-8. | Waist circumference and body mass index z scores were negatively correlated with thyroid-stimulating hormone levels (7–12 years old) after adjusting for age, sex, birthweight glucose, triglyceride and puberty status. |
|  | Eating behaviors | Healthier dietary pattern | Nutrients. 2016; 9(1): 4. | The dietary patterns, termed “healthy intake”, “animal food intake”, and “snack intake”, were identified at 7 years of age and were similar at 9 years of age. Changes in behaviors that improved the consumption of milk and dairy products or vegetables with every meal exhibited improved healthy intake pattern scores over 2 years. |
|  | Urinary bisphenol A | Liver enzymes | Environ Res. 2018; 161: 195-201. | The urinary bisphenol A concentrations at 7–9 years, but not those at 3–5 years, were positively associated with the serum levels of liver enzymes at 10–13 years of age. |
|  | Dietary patterns | Persistent organic pollutants in blood | Environ Pollut. 2018; 243(Pt A): 189-96. | The dioxin-like polychlorinated biphenyls (PCBs) pattern was characterized by a higher intake of yogurt, beverages, and fruit and a lower intake of grain, seaweeds, and processed meat. |
|  | Sex, age and height percentiles | blood pressure percentile curves (less than 10 years) | J Korean Med Sci. 2020; 35(12): e91. | We developed sex-, age-, and height-specific blood pressure reference values for non-overweight children aged 3–9 years. |
|  | Secondhand smoking | Behavioral problems | Sci Total Environ. 2020; 746: 141327. | The group with higher levels of cotinine at 5 years of age showed continuously higher total and external behavioral problem scores from ages 5–9. |
|  | Age | Trajectory model of blood pressure | Clin Hypertens. 2020; 26: 2. | A trajectory model of blood pressure was identified using repeated data from the age of 3 to 10 years. |
